# Detecting Tweets Containing Cannabidiol-Related COVID-19 Misinformation Using Transformer Language Models and Warning Letters From Food and Drug Administration: Content Analysis and Identification

**DOI:** 10.2196/38390

**Published:** 2023-01-23

**Authors:** Jason Turner, Mehmed Kantardzic, Rachel Vickers-Smith, Andrew G Brown

**Affiliations:** 1 Data Mining Lab Department of Computer Science and Engineering J B Speed School of Engineering, University of Louisville Louisville, KY United States; 2 Department of Epidemiology and Environmental Health College of Public Health University of Kentucky Lexington, KY United States; 3 Department of Criminology and Criminal Justice Northern Arizona University Tempe, AZ United States

**Keywords:** transformer, misinformation, deep learning, COVID-19, infodemic, pandemic, language model, health information, social media, Twitter, content analysis, cannabidiol, sentence vector, infodemiology

## Abstract

**Background:**

COVID-19 has introduced yet another opportunity to web-based sellers of loosely regulated substances, such as cannabidiol (CBD), to promote sales under false pretenses of curing the disease. Therefore, it has become necessary to innovate ways to identify such instances of misinformation.

**Objective:**

We sought to identify COVID-19 misinformation as it relates to the sales or promotion of CBD and used transformer-based language models to identify tweets semantically similar to quotes taken from known instances of misinformation. In this case, the known misinformation was the publicly available Warning Letters from Food and Drug Administration (FDA).

**Methods:**

We collected tweets using CBD- and COVID-19–related terms. Using a previously trained model, we extracted the tweets indicating commercialization and sales of CBD and annotated those containing COVID-19 misinformation according to the FDA definitions. We encoded the collection of tweets and misinformation quotes into sentence vectors and then calculated the cosine similarity between each quote and each tweet. This allowed us to establish a threshold to identify tweets that were making false claims regarding CBD and COVID-19 while minimizing the instances of false positives.

**Results:**

We demonstrated that by using quotes taken from Warning Letters issued by FDA to perpetrators of similar misinformation, we can identify semantically similar tweets that also contain misinformation. This was accomplished by identifying a cosine distance threshold between the sentence vectors of the Warning Letters and tweets.

**Conclusions:**

This research shows that commercial CBD or COVID-19 misinformation can potentially be identified and curbed using transformer-based language models and known prior instances of misinformation. Our approach functions without the need for labeled data, potentially reducing the time at which misinformation can be identified. Our approach shows promise in that it is easily adapted to identify other forms of misinformation related to loosely regulated substances.

## Introduction

### Background

The Food and Drug Administration (FDA) defines medical “misinformation” as the information that misleadingly represents a product as able to mitigate, prevent, treat, diagnose, or cure a condition or disease, such as COVID-19 [1]. Misinformation is prevalent on social networking platforms and is often seen in product advertising. Since early 2020, the COVID-19 pandemic has offered a new medical condition for advertisers and sellers to exploit, who seek to profit from the crisis at the expense of public health [2].

**Figure 1:**
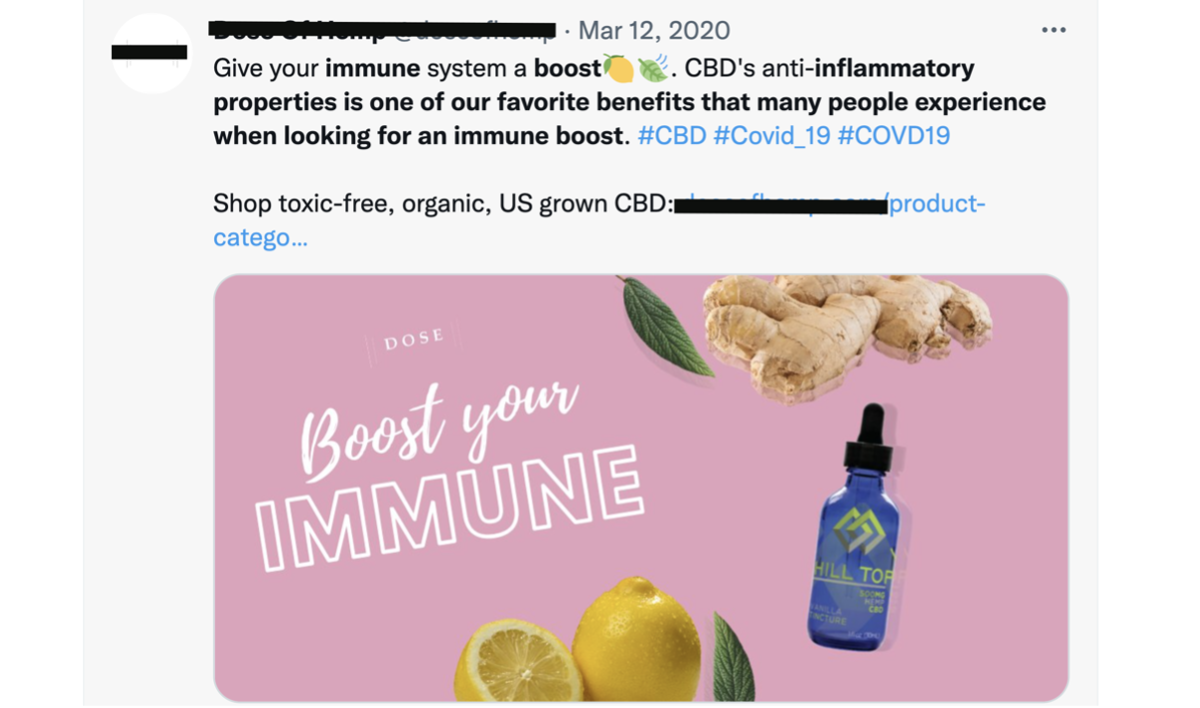
Example of a tweet promoting cannabidiol (CBD) as a prevention or treatment for COVID-19.

Although some misleading posts regarding COVID-19 have been about the virus’s origins or the effectiveness and safety of masks and vaccines [3-5], others have focused on false information about alternative products in treating or preventing COVID-19. For example, since the onset of the pandemic, some cannabidiol (CBD) sellers have claimed that CBD can prevent or cure COVID-19 (Figure 1). Preclinical studies have suggested that CBD could be effective in treating respiratory conditions and might confer cardioprotective, nephroprotective, hepatoprotective, neuroprotective, and anticonvulsant benefits [6]. Meanwhile, other evidence suggests that CBD could decrease one’s ability to fight infections, countering its potential clinical use as an anti-inflammatory agent [7]. Because CBD has not been thoroughly tested for efficacy and safety in treating COVID-19, its benefits are mostly considered unsubstantiated [8]. However, there is plentiful web-based marketing for CBD, touting these unverified benefits. This is not unique to CBD and COVID-19 and occurs with other loosely regulated substances (eg, kratom and herbal supplements) as well as other medical conditions such as Alzheimer disease, cancer, and autism. Although the consumption of CBD is typically well tolerated, misinformation about its effectiveness in treating COVID-19 has become widespread and poses a danger to public health [9,10]. Therefore, it is essential that this type of content be efficiently identified so that its potential harmful effects on public health can be minimized.

Misinformation has been shown to spread faster and further than accurate information on social media, and Twitter serves as an example of a social media site where misinformation about smoking products, drugs, vaccines, and diseases is abound [11,12]. Specifically regarding COVID-19, for example, in March 2020, a daily average of 46,000 misleading or inaccurate news posts appeared on Twitter in Italy alone [13]. In Iran, a rumor originating on social networks claimed that ingesting neat alcohol could cure COVID-19, resulting in hundreds of alcohol poisoning deaths [14]. Furthermore, influential Twitter users such as former President Donald J Trump and celebrity Joe Rogan suggested taking the antimalarial medications chloroquine or hydroxychloroquine to treat COVID-19, even though there has never been scientific evidence to support these claims [15-17]. Although the United States FDA warned against improper consumption of these substances in July 2020, there were still dozens of documented deaths and poisonings associated with their use, including at least one person who ingested fish tank cleaner containing chloroquine after being influenced by misinformation on Twitter [18-20]. Not only has misinformation dissuaded consumers from seeking effective treatments but also it has encouraged the use of dangerous and unfounded *treatments*.

Prior research has shown some success in using supervised and unsupervised machine learning techniques to detect and explore COVID-19 misinformation on social media platforms such as Twitter [21-24] and YouTube [25]. Although some studies used an annotated data approach [21,24,26], it was noted that the rigor and costs of this process could preclude its widespread utility [21]. One study used an unsupervised topic model to examine inaccurate information about vaping CBD and COVID-19 [27], which is a more cost-effective technique than annotation, but is a less explicit way to identify misinformation than supervised approaches. Deep learning [23,28] and transformer language models [22,23,25,29] are the most commonly used techniques, likely because of their efficiency and highly advanced ability to interpret and understand natural language, which is key to examining content on social media.

This study leverages the expertise of the FDA, the regulatory body of the safety and efficacy of numerous products intended for human or animal use in the United States, to define misinformation regarding CBD and COVID-19 using Warning Letters as a gold standard. When the FDA becomes aware that a company has violated FDA regulations, they often issue Warning Letters to the company that outline the nature of the violation (eg, problems with claims about a product and incorrect directions for use), corrective action, and a timeframe [30]. The FDA then follows up to verify whether the company has completed the corrective action, and if not, the FDA may enact regulatory actions such as seizure or civil penalties [31]. These Warning Letters are made available to the public on the FDA’s website [32]. By using verbatim quotes taken from relevant FDA Warning Letters and transformer language models, we propose an efficient and consistent approach to identify tweets that contain false claims regarding CBD and COVID-19.

### Objectives

This study has two primary objectives: (1) to examine misinformation concerning CBD and COVID-19 disseminated by Twitter advertisers and (2) to propose a framework that helps identify CBD- and COVID-19–related misinformation in tweets more efficiently and can also be easily modified to detect misinformation in advertisements about other substances and conditions.

Traditional text classification requires labeled data for model training and testing, which can be both time consuming and expensive. Transformer-based language models have shown promising results in various semantic textual similarity tasks, including in the medical domain [33].

## Methods

### Ethics Approval

This study leverages publicly available data and was registered as approved by the University of Louisville Institutional Review Board (approval protocol 20.1122).

### Collecting and Annotating Tweets

We used the Snscrape Python package to collect English language tweets from the Twitter website from January 1, 2020, to April 28, 2021, by searching for tweets using the following keywords: *CBD or cannabidiol*, and *COVID-19, COVID, corona* [34]. Although this method does not provide full access to Twitter’s past data, it does afford the ability to collect thousands of historical tweets retrospectively after an unexpected event has occurred, such as the pandemic. Using this method of historical tweet searching, we were able to collect 37,526 tweets over a 484-day period.

To extract the commercial CBD tweets referencing COVID-19, we used a model previously developed by our group [35]. The model was trained on an earlier collection of CBD tweets to identify those that reflected commercial sales, promotion, and marketing of CBD. Applying the commercial CBD tweet classifier to the historical tweet collection resulted in 4937 tweets that were classified as commercial CBD tweets referencing COVID-19. We evaluated the performance of this model that identified commercial CBD tweets from the CBD or COVID-19 collection of tweets by annotating a random sample of classified 250 commercial CBD or COVID-19 tweets and 250 noncommercial CBD or COVID-19 tweets. We observed an improvement in the commercial tweet classification performance, with precision, recall, accuracy, and *F*_1_-scores of 0.95 (Table 1).

**Table 1:** Performance of 2019 commercial cannabidiol (CBD) classifier in differentiating tweets from 2020 to 2021 (N=500).^a^

	Precision	Recall	*F*_1_-score	Random sample
Noncommercial CBD	0.95	0.95	0.95	249
Commercial CBD	0.95	0.95	0.95	251

^a^Accuracy of the model: 0.95.

The United States FDA has warned CBD advertisers and sellers about promoting CBD as a treatment or cure for several conditions, including COVID-19. These Warning Letters inform the advertiser that CBD is not an approved treatment or prevention for the condition that the advertiser mentions, warn the advertiser that further action will be taken if this style of misleading advertising continues, and include the exact misleading quote taken from the advertisement. This list of letters continues to grow as the FDA becomes aware of more advertisers making false claims about the medical benefits of CBD. We drew directly from the FDA Warning Letters about CBD and COVID-19 to understand the types of statements that the FDA had flagged as misinformation.

We used the statements from these FDA Warning Letters to identify and label the CBD tweets containing COVID-19–related misinformation. Instead of using our annotated set of tweets to train and test a tweet classification model, we used them to establish the relationship of text similarity between the misinformation tweets and the statements in the FDA Warning Letters via the transformer language model. The transformer architecture was introduced to address some of the shortcomings of the recurrent neural networks in tasks such as language translation [36].

**Figure 2:**
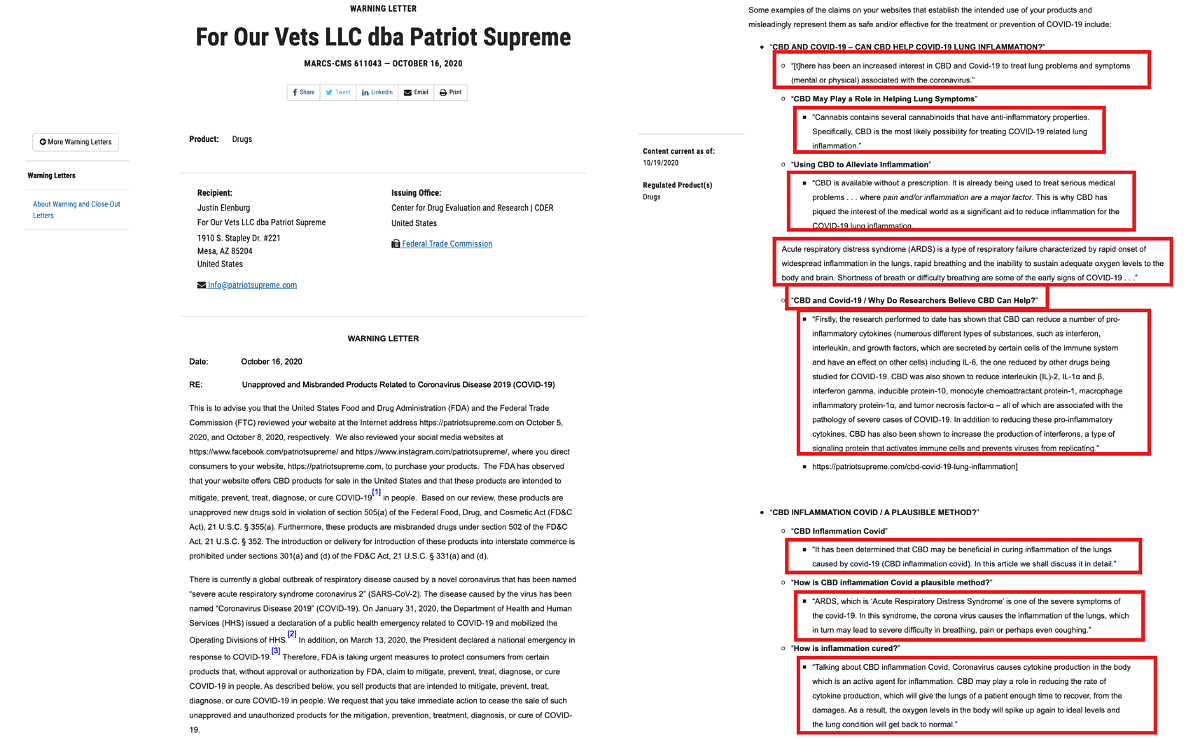
Example Warning Letter taken from the Food and Drug Administration (FDA) website.

Because the FDA’s CBD or COVID-19 Warning Letters did not include CBD-infused hand sanitizers, we included the Warning Letters sent to advertisers of nonalcohol and essential oil sanitizer products that had made false claims as guidance in our misinformation annotation process [37-43]. Figure 2 is an example of one of the Warning Letters issued by the FDA about misinformation surrounding the use of CBD for treating COVID-19.

The extracted commercial CBD or COVID-19 tweets were annotated for misinformation (yes or no), according to the FDA’s definition of misinformation, by 3 university-trained, nonmedical, professional annotators; discrepancies were decided by a majority vote to determine the overall label of the tweet. Before labeling the tweets, we reviewed several FDA Warning Letters with the annotators so that the annotators were familiar with what the FDA considered a misleading statement. Because tweets are relatively short in length, if a tweet contained any misinformation related to CBD and COVID-19, the entire tweet was considered positive for misinformation. There were no noticeable discrepancies among the annotators, and they had an intercoder agreement of 91%.

The Warning Letters on the FDA website provided some of the quotes that sellers used in their advertisements that the FDA deemed misleading. Some examples of these letters are listed in Textbox 1. Along with the FDA’s definition of misinformation, these quotes provided guidelines for annotating the collection of tweets as to whether they contained misinformation. Textbox 2 displays selected examples of the nonmisinformation and misinformation tweets encountered during the annotation process.

**Five example quotes from Warning Letters issued by Food and Drug Administration (FDA) related to cannabidiol (CBD) and COVID-19 misinformation.**

- “Firstly, the research performed to date has shown that CBD can reduce a number of pro-inflammatory cytokines (numerous different types of substances, such as interferon, interleukin, and growth factors, which are secreted by certain cells of the immune system and have an effect on other cells) including IL-6, the one reduced by other drugs being studied for COVID-19. CBD was also shown to reduce interleukin (IL)-2, IL-1*α* and *β*, interferon gamma, inducible protein-10, monocyte chemoattractant protein-1, macrophage inflammatory protein-1*α*, and tumor necrosis factor-*α* – all of which are associated with the pathology of severe cases of COVID-19. In addition to reducing these pro-inflammatory cytokines, CBD has also been shown to increase the production of interferons, a type of signaling protein that activates immune cells and prevents viruses from replicating”
- “There has been an increased interest in CBD and Covid-19 to treat lung problems and symptoms (mental or physical) associated with the coronavirus”
- “CBD oil may help to prevent getting infected by strengthening your immune system. It has also been proven to offer relief to some of the symptoms”
- “By using CBD oil, you can keep inflammation at bay, retain a healthy or even higher than average white blood cell count, stay calm and relaxed (which is best for a strong immune system), and prevent catching a virus or infection beforehand”
- “Is CBD an Anti-Viral Agent for Coronavirus, Influenza, MERS, and Sars Plus Key Antiviral Supplements?”

**Examples of annotated commercial cannabidiol (CBD) or COVID-19 tweets.**

- Misinformation
  “#CBD is readily available for anyone who want to build-up their immune to help guard against the #coronavirus. It’s your responsibility to protect &; take care of yourself, not the government. Order Now”
  “I’ve ordered a 4 month supply of #CBD to help fortify my immune system &; guard against the #CoronaVirusUpdates #COVID19. What have you done to protect yourself? Order Now”
  “#COVID19 attacks the inside of body/lungs which are internal so topical solutions will not mitigate what's happening inside your body/lungs. Ask me about #CBD. #cbdoil <MASKED-URL>”
  “#CBDL Could Double On Product Launch News. CBD Hand Sanitizer Could Help Stop Spread Of Covid-19. [Read Now] LINK #USA #Stocks #Bonds #Equities #Gold #Silver #Bitcoin #CryptoCurrency #Investing #Trading #Options #1Author #USStocks #StockMarket”
- Nonmisinformation
  “Could CBD offer treatment options for COVID-19? Read more at <MASKED-URL> Global Go does not endorse the use of any product for medicinal purposes. Please consult with a physician before using any such products. #CBDtrials #hempresearch #hempnews #covid19”
  “First day of the week... First day of the month of June! Would you like to try something new? #Covid19 #SanFrancisco #SanFranciscobayArea” #helpingthecommunity #Realestate #HartFordproperties #CBD Source:
  “Online sales for cannabidiol (#CBD) products continue to #flourish despite in-store slowdowns amid the COVID-19 #pandemic. #CSPDailyNews”
  “New post (Iowa Down To One Medical CBD Manufacturer Due To COVID-19 Pandemic) has been published on Buy Premium CBD and CBG Products | 100% Natural Cannabinol Store | Buy CBD Oils, Gummies, Topicals, Pet CBD and more.”

### Misinformation Search

The transformer follows an encoder-decoder structure wherein the encoder converts the text input into a vector representation. There is one vector per word in the sequence; the value of each vector representation of each word is partially based on the nearby terms surrounding the word, which provides the context. The decoder portion of the transformer architecture is similar to the encoder but can convert a vector into a sequence of text. The original Bidirectional Encoder Representations from Transformers (BERT) model was introduced by Devlin et al [44] at Google in 2018. The BERT model is trained on the natural language processing tasks of masked language modeling on English Wikipedia and the Brown Corpus texts and it predicts the missing word in a sentence and performs next-sentence prediction. Masked language modeling allows the BERT model to understand the relationships between words, whereas next-sentence prediction allows the BERT model to understand the relationships between sentences.

In this study, we used a transformer language model to encode each of the commercial CBD tweets that we collected. Specifically, we used the Sentence-T5 model because it is a state-of-the-art language model that has outperformed other models in semantic textual similarity [45]. We also computed the encoding for each statement containing misinformation taken from the FDA website into vectors of size 768. We then calculated the cosine similarity (Equation 1) between the encoding vectors of tweets (A) and the encoding vectors of the misinformation quotes taken from the FDA Warning Letters (B).



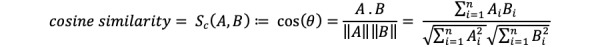



**Figure 3:**
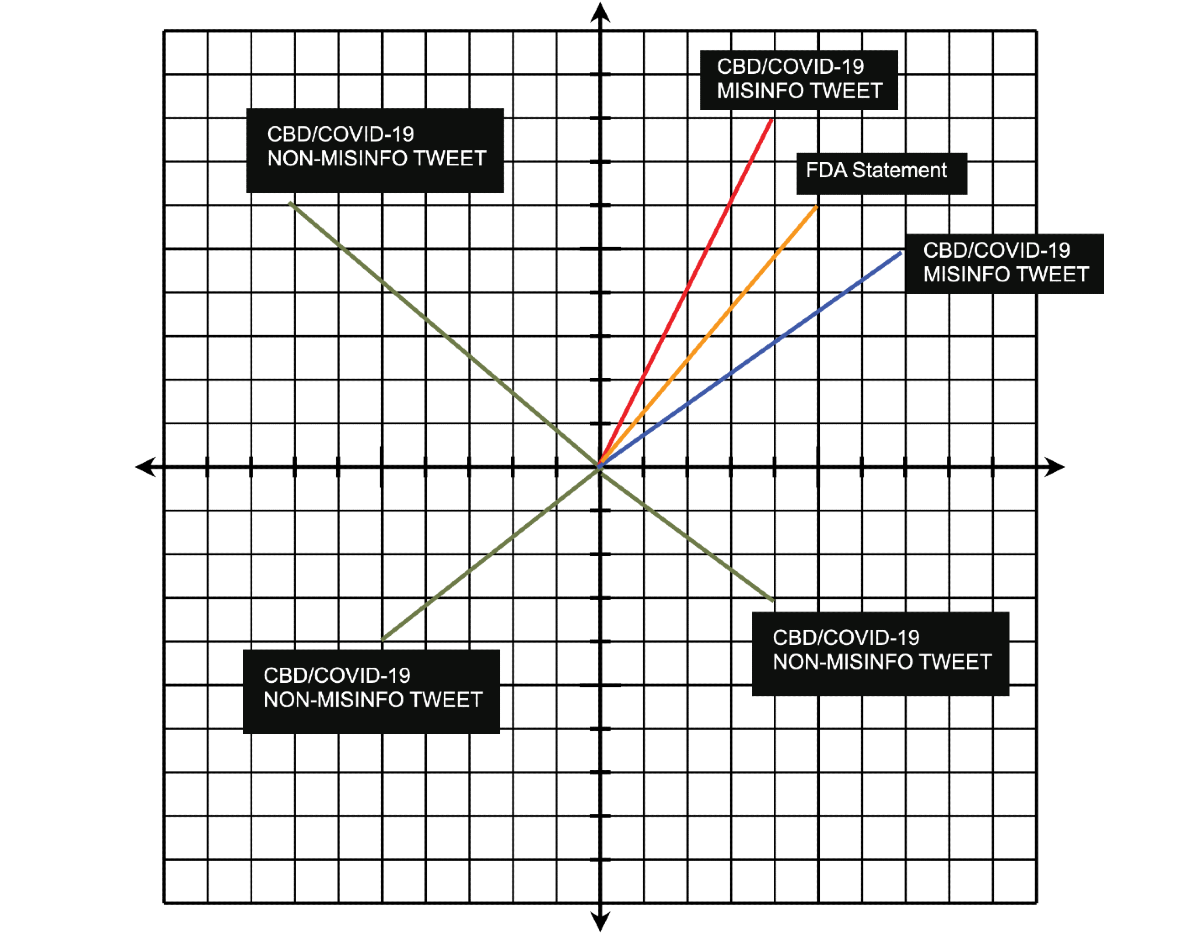
Representation of contextual similarity between cannabidiol (CBD) tweets and quotes from Food and Drug Administration (FDA) Warning Letters.

Figure 3 provides a theoretical illustration of how we isolated tweets that made false claims using the quotes taken from the FDA Warning Letters to find contextual similarities using cosine similarity. This example shows that when the tweets and quotes from Warning Letters are encoded into vectors, tweets containing CBD or COVID-19 misinformation will be closer in cosine distance to the FDA quotes than tweets without similar false claims about CBD and COVID-19. Using cosine similarity as the distance of similarity, we expected that the shortest cosine distances would contain more misinformation, that is, tweets that were contextually similar to the FDA’s samples. Conversely, we expected the tweets with the longest cosine distances to contain less misinformation. Using these points, we identified a threshold at which we could confidently identify sets of tweets that mostly contained misinformation because of their semantic similarity to an established example of misinformation. The optimal threshold should be the point at which the maximum number of tweets deemed as misinformation is captured while minimizing the number of false positives (ie, tweets that do not contain misinformation) being captured.

## Results

### Analysis of Misinformation

After annotating the tweets for misinformation, we observed that approximately 19% (938/4937) of the tweets contained misinformation related to both CBD and COVID-19. Table 2 shows some of the terms most frequently associated with CBD or COVID-19 misinformation. Aside from CBD-related terms, terms such as “help,” “boost,” “health,” “virus,” “sanitizer,” and “immune system” were among the most frequently occurring terms. We observed an increase in CBD or COVID-19 conversations beginning in February 2020. Misinformation related to CBD and COVID-19 peaked in March 2020; although it appeared to taper down, it did not stop.

**Table 2:** Top n-grams in the cannabidiol or COVID-19 misinformation tweets.

Term	Frequency
immune	257
cbdoil	244
system	187
immune system	181
products	157
help	152
hemp	139
health	129
virus	127
new	127
cbd cbdoil	125
natural	119
wellness	114
boost	112
oil	99
hand	96
sanitizer	92
cbdproducts	88
cbd products	85
hand sanitizer	83
use	81
immunity	78

### Detecting Misinformation

Using 27 misleading quotes taken from the FDA Warning Letters and converting them into vector form and then converting each tweet into vector form, we calculated the cosine similarity. Using the cosine distance, we then counted the number of tweets that were labeled as misinformation compared with the tweets that were considered nonmisinformation. We observed that the nearest tweets indeed contained misinformation, whereas the most distant tweets did not (Table 3).

**Table 3:** Measuring the cosine distance between sentence vectors of statement 0 and the tweets.

Tweet	Position	Misinformation			Cosine distance
		Yes	No		
“With the growing concern of the COVID-19 virus we understand the importance of boosting the immune system. CBD^a^ is a natural way to do that. The Healing Leaf wants to help make CBD more available and lessen costs for those interested. Please come in or call and place your orders!” <MASKED-URL>	Most similar	✓		0.069577	
“CBD may reduce cytokine storm and inflammation in COVID-19\n” <MASKED-URL>	Second most similar	✓		0.077852	
“They snuck a shipping ban on vape products into the last covid relief bill. All vape products. CBD, nicotine, delta 8, doesn’t matter.”	Second most distant		✓	0.296933	
“Final point: When COVID hit, we LOWERED our prices and never put them back up. Funny how reddit and Review haters never use their real names and 9/10 when you find out their real name - they run for the hills or perhaps just back to Mam’s house and their keyboard...”	Most distant		✓	0.297551	

^a^CBD: cannabidiol.

**Figure 4:**
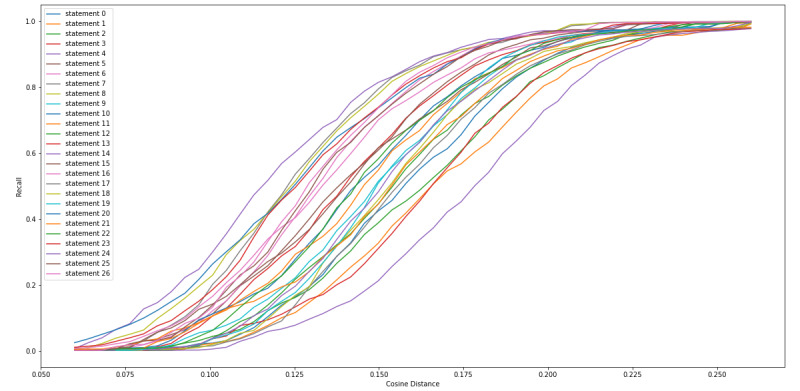
Cosine distance versus proportion of misinformation tweets captured.

**Figure 5:**
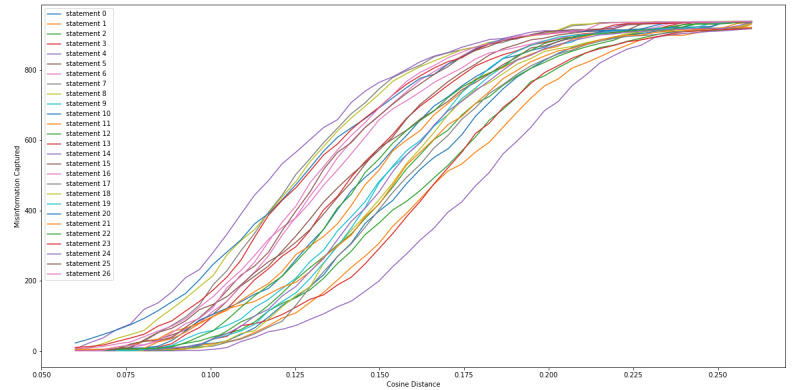
Cosine distance versus number of misinformation tweets captured.

**Figure 6:**
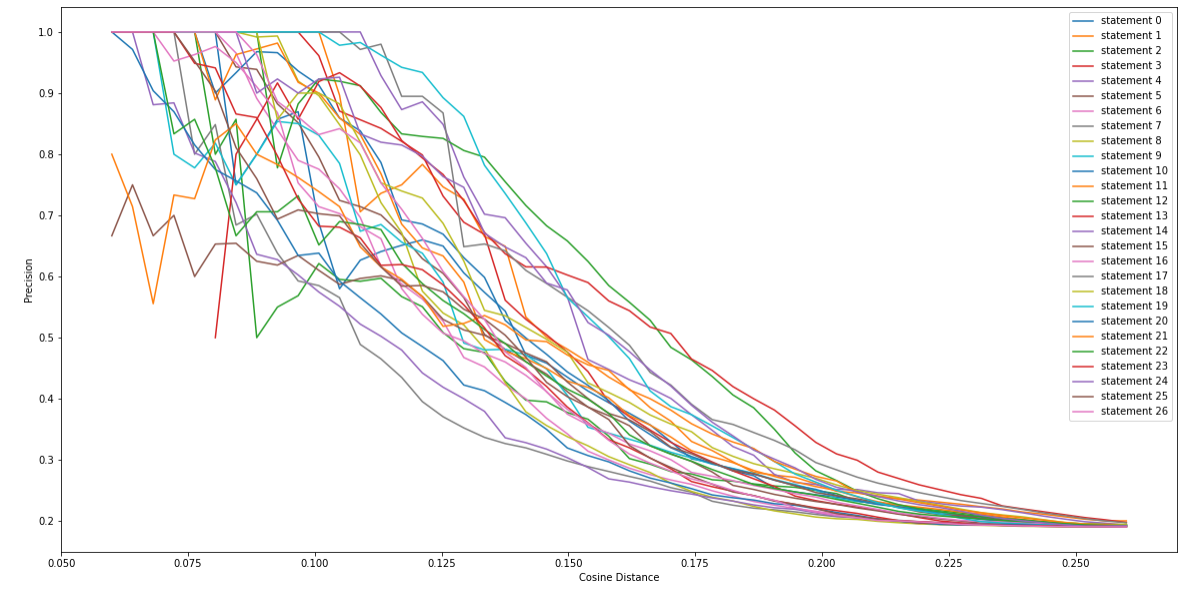
Cosine distance versus misinformation precision of tweets captured.

We calculated the cosine distance between the sentence vector of each of the 27 statements that we extracted from the FDA Warning Letters, along with each of the tweets, so that we could determine a cosine distance threshold in which we collected the most tweets that contained CBD or COVID-19 misinformation while minimizing false positives (non-CBD or COVID-19 misinformation). Figures 4 and 5 show a consistent observation that as the cosine distance increased, the percentage (recall) and frequency of the tweets, respectively, containing misinformation increased. Figure 6 indicates that as the cosine distance increased, the precision of the tweets containing misinformation decreased. However, if the cosine distance was too small, few to no misinformation tweets were captured, and not all FDA statements performed equally in capturing misinformation tweets.

**Figure 7:**
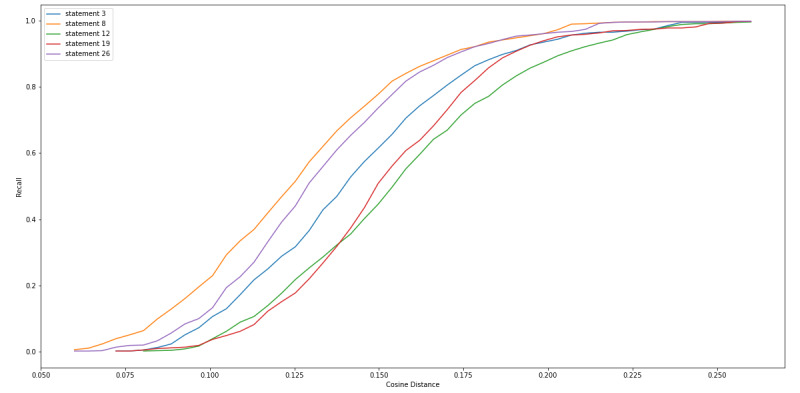
Cosine distance versus percentage of misinformation tweets captured by top 5 performing Food and Drug Administration (FDA) statements, which captured large amount of misinformation with high precision.

**Figure 8:**
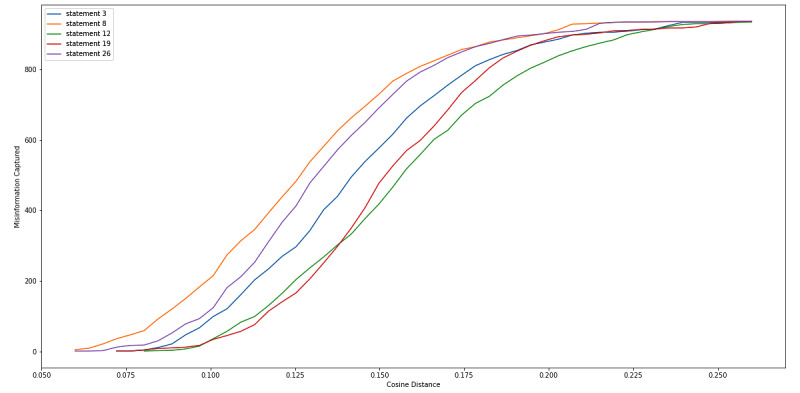
Cosine distance versus number of misinformation tweets captured by top 5 performing Food and Drug Administration (FDA) statements, which captured large amount of misinformation with high precision.

**Figure 9:**
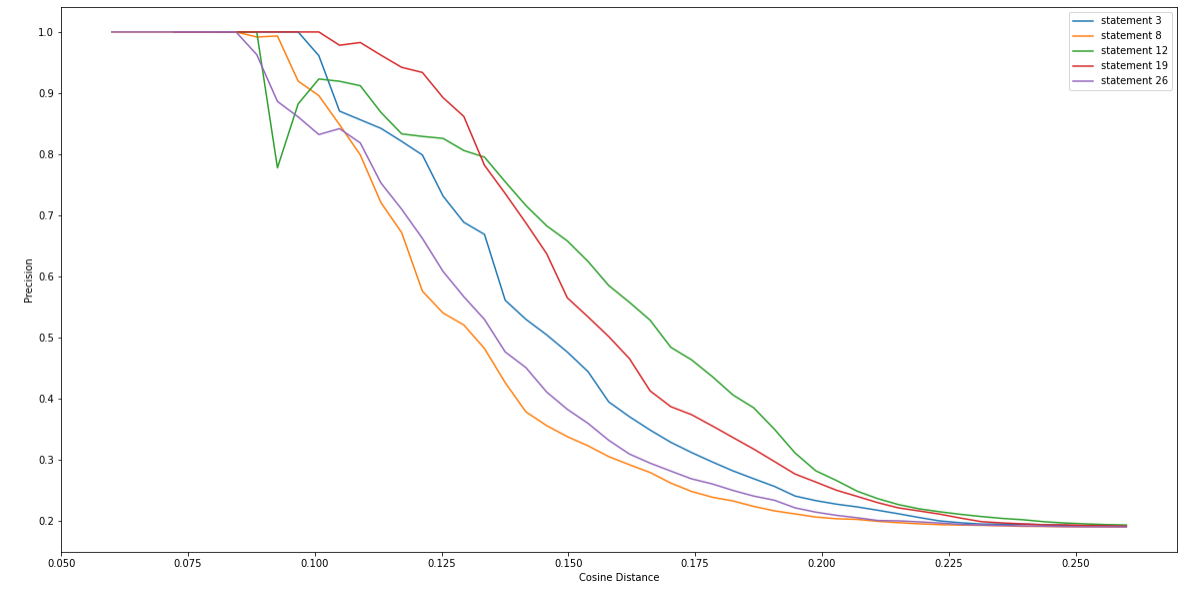
Cosine distance versus misinformation precision of tweets captured.

We also examined the top 5 FDA statements that captured large amounts of misinformation with high precision (Textbox 3). Figures 7-9 are equivalent to Figures 4-6 but include only the statements displayed in Textbox 3. From these figures, we can see that at a cosine distance between 0.10 and 0.13, we were able to capture between 21.9% (206/938) and 28.9% (272/938) of the misinformation tweets with a precision of above 80%. Specifically, statement 8 was able to capture 34.4% (323/938) of tweets at a cosine distance of 0.105, and 84.8% (274/938) of these tweets were labeled as misinformation. The 274 captured tweets represented 29.2% of the 938 misinformation in our data set.

**Food and Drug Administration (FDA) statements that captured the largest amount of misinformation with the highest amount of precision.**

- Statement 3
  “Firstly, the research performed to date has shown that CBD can reduce a number of pro-inflammatory cytokines (numerous different types of substances, such as interferon, interleukin, and growth factors, which are secreted by certain cells of the immune system and have an effect on other cells) including IL-6, the one reduced by other drugs being studied for COVID-19. CBD was also shown to reduce interleukin (IL)-2, IL-1*α* and *β*, interferon gamma, inducible protein-10, monocyte chemoattractant protein-1, macrophage inflammatory protein-1α, and tumor necrosis factor-*α* – all of which are associated with the pathology of severe cases of COVID-19. In addition to reducing these pro-inflammatory cytokines, CBD has also been shown to increase the production of interferons, a type of signaling protein that activates immune cells and prevents viruses from replicating”
- Statement 8
  “DML CBD: Immune Boost Pack... ALERT: There is no cure or treatment for COVID19. With this in mind, many doctors claim the best defense is to boost the body’s immune system. DML CBD aims to help our customers in an attempt to boost the immune system... WHY TO BUY THE BOOST PACK: Studies suggest that CBD can help fight off inflammation, boost the immune system, and help battle against certain harmful bacteria. Some research suggests it can help suppress the cytokine storm inside the body that can cause great illness and sometimes death... NOTE: The cytokine storm is often triggered in patients with COVID19. Please note there is no proven cure or treatment for COVID19... There has never been a more important time than to boost your immune system. To help our customers get a full CBD experience that aims to boost your immune system, we offer the ‘DML CBD Immune Boost’ package...”
- Statement 12
  “What is COVID-19? Coronavirus is referred to as a novel cause for viral pneumonia because it’s a virus we haven’t seen before and have developed no immunity to... What Happens If You Get Infected and What Can Help? ...Regardless of the shape you’re in at this moment, there may be ways you can prepare and protect your body from developing a more severe response to infection. Explore the solutions included in NoronaPak below! [graphic with the following text] ‘Selenium, Cannabidiol (CBD), Vitamin-C, Zinc, Vitamin-D, N-Acetylcysteine’... Supplementation with selenium results in changes in the gene expression that is required for protein biosynthesis in lymphocytes, the infection-fighting cells that are crucial to the immune system being able to identify infection and mount an immune response... Selenium is not only important in boosting the immunity of the individual but also to slow the development of more virulent strains of some viral pathogens... CBD may suppress the productions of cytokines in the setting of infection”
- Statement 12
  “In the wake of the current epidemic, it is now more important than ever to keep your immune system as healthy as can be... Here are 5 key ways to strengthen your immune system during the outbreak...Take supplements such as CBD”
- Statement 26
  “Crush Corona... While scientists around the world are working 24/7 to develop a COVID-19 vaccine, it will take many more months of testing before it’s approved and available. However, there’s something you can do right now to strengthen your immune system. Take CBD... CBD can help keep your immune system at the stop of its game... We want everyone to take CBD and take advantage of its potential to help prepare your body to fight a coronavirus infection. So, we’re making all of our products more affordable”

### Application to Other Contexts

**Figure 10:**
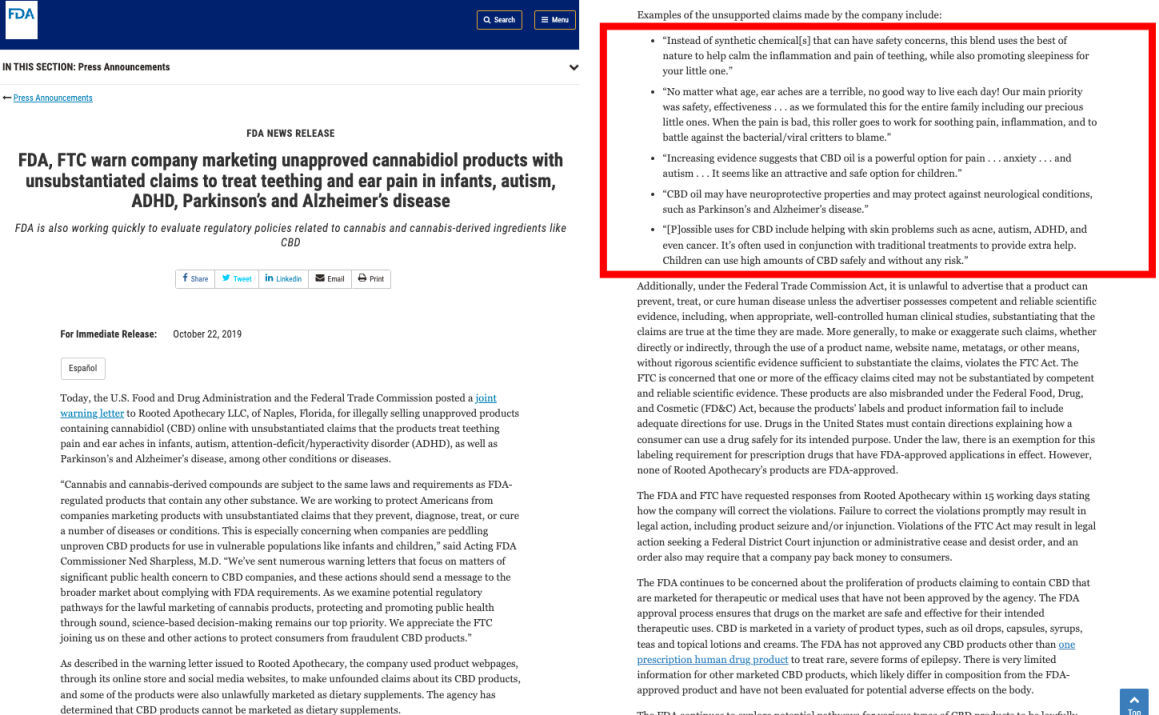
Warning Letter taken from Food and Drug Administration (FDA) website regarding cannabidiol (CBD) as a treat for teething, autism, attention deficit hyperactivity disorder (ADHD), and Alzheimer disease.

To further demonstrate the flexibility of our methods, we applied it to a corpus of tweets collected in 2019 using only the terms “CBD” and “cannabidiol,” and misleading quotes from FDA Warning Letters regarding autism and Alzheimer disease (Figure 10). As shown in Table 4, we observed that the tweets most similar to the misinformation samples suggested that CBD could alleviate the symptoms of autism and Alzheimer disease. The most distant tweets did not make false claims about CBD’s ability to treat those conditions.

**Table 4:** Most similar and most distant (in cosine similarity) tweets to misinformation quotes regarding Alzheimer disease.

FDA^a^ misinformation quote	Most similar tweet	Most distant tweet
“CBD^b^ oil may have neuroprotective properties and may protect against neurological conditions, such as Parkinson’s and Alzheimer’s disease”	@<MASKED-USER> @<MASKED-USER> @<MASKED-USER> @<MASKED-USER> @<MASKED-USER> “On the up side, some evidence suggests THC^c^ and CBD may be neuroprotective, so there’s a rationale for some MMJ for you. Alzheimer’s prophylaxis”	@<MASKED-USER> “do not believe he was the co-owner and while the store is definitely shitty, the sign did not say CBD cures autism”
“Possible uses for CBD include helping with skin problems such as acne, autism, ADHD^d^, and even cancer. It’s often used in conjunction with traditional treatments to provide extra help. Children can use high amounts of CBD safely and without any risk.”	“CBD is recommended as a treatment for conditions such as seizures, depression and anxiety, and symptoms such as sleeplessness, inflammation, acne, and pain. It has also proven to be effective in treating autistic children. \nSource: <MASKED-URL> <MASKED-URL>”	“4) Sydney is demonstrating that she understands what effectual customer demands are, which is going straight to the source to demand change. She did not see a phone number she could call on the sign that promoted CBD as a cure-all for autism, so she went in to ask for one”

^a^FDA: Food and Drug Administration.

^b^CBD: cannabidiol.

^c^THC: tetrahydrocannabinol.

^d^ADHD: attention deficit hyperactivity disorder.

## Discussion

### Principal Findings

COVID-19–related misinformation can have fatal consequences [46]. Those consuming this misinformation may have misconceptions about how the virus is transmitted, disease symptoms, or health effects; they may communicate misinformation to others who subsequently spread it and put themselves and others at risk. Although there have been some preliminary studies on the benefits of cannabis in treating the symptoms of COVID-19, findings are uncertain, and at the time of this writing, cannabis is not an approved treatment by the FDA [47]. Therefore, claiming that CBD products can unequivocally treat or prevent COVID-19 is a federal violation in the United States, where the FDA has made numerous attempts to warn web-based retailers making false claims about their products [1]. Our study demonstrates how FDA Warning Letters can be used with transformer language models to identify tweets containing misinformation that are semantically similar to Warning Letters. Our approach reduces the time, labor, and potential monetary costs of other text classification methods. To our knowledge, this is the first study to use FDA Warning Letters as a foundation for identifying misinformation, particularly as it relates to CBD and COVID-19, in web-based social networks.

Transformer language models, such as the one used herein, are powerful tools that have been used for a number of purposes, such as translation [48,49] and text classification [50,51], and have even been used to generate descriptions of images in text [52]. Because of their ability to understand and summarize natural language, several studies have used these models to identify web-based misinformation, which often includes nuanced language and requires techniques that recognize not only semantic but also contextual similarities. Kumar et al [22] used Twitter to build a multilabel tweet classifier system in their study using a RoBERTa-large transformer language model to identify COVID-19–related misinformation. Their model could identify 4 classes: irrelevant, conspiracy, true information, and false information, and it achieved an *F*_1_-score of 76%. Although this study focused on any class of misinformation that was identified in the FDA Warning Letters, future studies could further classify the letters by type and evaluate the accuracy of our approach.

Although this work was built on only Twitter platform, it is possible that it could also be applied to other social network platforms. Serrano et al [25] used transformer-based language models to identify YouTube videos containing COVID-19–related misinformation via comments posted on the videos. They built a text classifier to identify conspiracy-related content and concluded that YouTube videos containing misinformation were accompanied by user comments with a high percentage of conspiracy-related content [25]. Future studies can assess the performance of our method on other platforms.

In a recent approach to concept drift (changes in data and meaning over time) in Twitter data streams, Bechini et al [53] trained a semantic-based classifier using the BERT language model to examine the change in opinions about vaccines within a corpus of Italian tweets; this model outperformed other strategies, such as retraining the ensemble approaches. This suggests that transformer-based models, such as the one described herein, for identifying commercial tweets can be resilient over time. In addition, given more extensive (eg, “firehose” access) and future access to Twitter data, our misinformation tweet classifier could identify newer and current tweets that were not included in our data set, as well as identify tweets making similar violations in near real time. Furthermore, as previously noted, this technology can be applied to other forms of misinformation that threaten public health and safety.

Our approach to identifying tweets that make false claims about CBD and COVID-19 used quotes extracted from FDA Warning Letters to identify tweets that are semantically and contextually similar based on the cosine distance of sentence vectors. Compared with the approaches that require a large amount of data annotation, this substantially reduces the time required to identify the tweets making false health-related claims and flag them with a high amount of confidence. This is attributed to 2 factors: we used minimal manually annotated data for validation purposes and we used a simple calculation of cosine similarity between tweets and quotes.

Our study not only illustrates the scope of misleading information about CBD and COVID-19 in particular but also demonstrates an efficient and affordable approach to identifying other instances of this widespread problem—an approach that can be used by government entities, social networks, and message board administrators concerned with minimizing false advertising and misinformation and the potential threats they pose.

### Limitations

This study had several limitations. First, it was built only on the Twitter social networking platform. In addition, although we did not explicitly acknowledge “bots” on Twitter, this was implicitly addressed during the annotation process where the tweets that appeared to be machine-generated were not considered cases of commercial CBD or misinformation. In addition, this model was trained on a collection of CBD tweets from before the COVID-19 pandemic, that is, before words like “COVID,” “COVID-19,” “corona,” and “coronavirus” would have been associated with “CBD” and “cannabidiol.” In this case, we applied a CBD commercial tweet classifier that was trained on tweets that were primarily authored in 2019 to a collection of tweets between 2020 and 2021. Although we did observe satisfactory results in testing this model’s extraction of commercial CBD tweets that also mentioned COVID-19, we acknowledge that concept drift is always a potential factor in classifying streaming and should always be considered.

### Conclusions

There is a clear and pronounced advertising presence on Twitter of loosely regulated substances touted to treat COVID-19, although this type of self-treatment lacks evidentiary support. Twitter is a medium known for rapid spread of medical misinformation, perhaps especially concerning substances like CBD [54-57]. The COVID-19 pandemic has been yet another opportunity for CBD marketers and sellers to mislead the public about CBD’s role in treating or preventing the disease. Illegitimate web-based CBD sellers pose a public threat by spreading misinformation, selling unregulated products, and generally sidestepping regulations. Our approach to addressing this issue identified a semantic relationship between tweets containing false claims about CBD in treating COVID-19 and FDA Warning Letters. Using transformer language models and quotes from FDA Warning Letters to other CBD advertisers, this framework can be easily adapted to find misinformation about other conditions and substances, thereby potentially serving a crucial purpose in benefiting public health.

## Abbreviations

BERT: Bidirectional Encoder Representations from Transformers
CBD: cannabidiol
FDA: Food and Drug Administration
